# Potential Health Benefits of Plant Food-Derived Bioactive Components: An Overview

**DOI:** 10.3390/foods10040839

**Published:** 2021-04-12

**Authors:** Mrinal Samtiya, Rotimi E. Aluko, Tejpal Dhewa, José Manuel Moreno-Rojas

**Affiliations:** 1 Department of Nutrition Biology, School of Interdisciplinary and Applied Sciences, Central University of Haryana, Mahendergarh, Haryana 123031, India; mrinalsamtiya@gmail.com; 2 Department of Food and Human Nutritional Sciences, University of Manitoba, Winnipeg, MB R3T 2N2, Canada; rotimi.aluko@umanitoba.ca; 3 Department of Food Science and Health, Andalusian Institute of Agricultural and Fisheries Research and Training (IFAPA), Alameda del Obispo, Avda. Menéndez Pidal, SN, 14004 Córdoba, Spain

**Keywords:** plant foods, bioactive components, antioxidants, polyphenols, anti-inflammatory, chronic diseases, human health, gut health

## Abstract

Plant foods are consumed worldwide due to their immense energy density and nutritive value. Their consumption has been following an increasing trend due to several metabolic disorders linked to non-vegetarian diets. In addition to their nutritive value, plant foods contain several bioactive constituents that have been shown to possess health-promoting properties. Plant-derived bioactive compounds, such as biologically active proteins, polyphenols, phytosterols, biogenic amines, carotenoids, etc., have been reported to be beneficial for human health, for instance in cases of cancer, cardiovascular diseases, and diabetes, as well as for people with gut, immune function, and neurodegenerative disorders. Previous studies have reported that bioactive components possess antioxidative, anti-inflammatory, and immunomodulatory properties, in addition to improving intestinal barrier functioning etc., which contribute to their ability to mitigate the pathological impact of various human diseases. This review describes the bioactive components derived from fruit, vegetables, cereals, and other plant sources with health promoting attributes, and the mechanisms responsible for the bioactive properties of some of these plant components. This review mainly compiles the potential of food derived bioactive compounds, providing information for researchers that may be valuable for devising future strategies such as choosing promising bioactive ingredients to make functional foods for various non-communicable disorders.

## 1. Introduction

**Figure 1:**
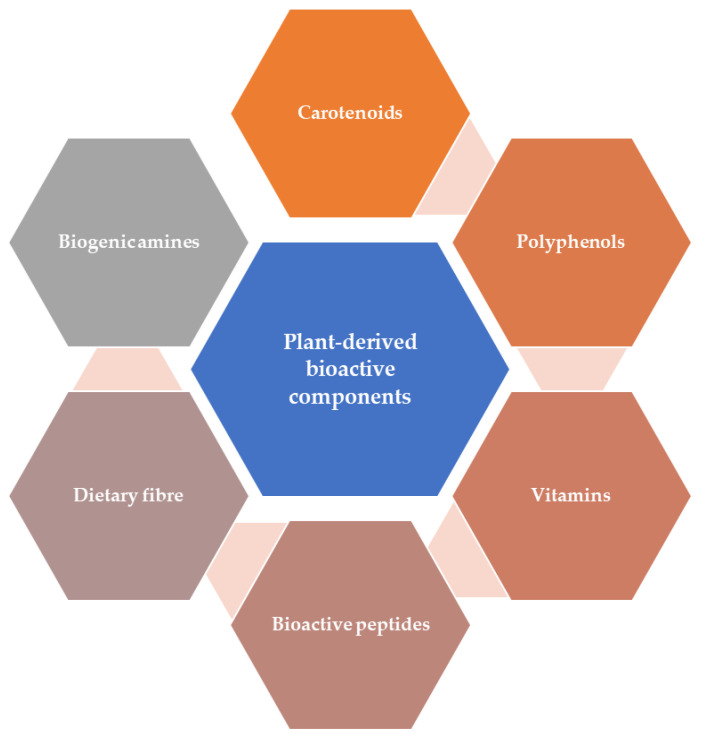
Brief overview of plant-derived bioactive compounds.

Food delivers several nutrients that are essential for life as well as many molecules or components with bioactive properties involved in health improvement and disease protection. Guidelines for a healthy diet recommend high intakes of plant-based foods such as eating at least 400 g of fruits and vegetables on a daily basis [1]. Plants contain minerals that are necessary components of a healthy human diet, in addition to several primary and secondary metabolites that influence nutrition and human health [2]. Secondary metabolites are not crucial for the general development or functioning of plants, but these compounds present biological activity that makes them very useful as ingredients to formulate traditional and modern medicines. Fruits and vegetables are rich sources of micronutrients (magnesium, calcium, and potassium) and bioactive components (including non-nutrients) consisting of phytochemicals such as polyphenols, dietary fiber, carotenoids, and vitamins. Figure 1 shows a brief overview of plant-derived bioactive compounds.

It has been established that more than 5000 different phytochemical compounds are present in grains, vegetables and fruits, though many more remain unidentified [3]. Enormously advantageous attributes of whole grains, vegetables, and fruits have been associated with bioactive non-nutritional chemical components commonly known as phytochemicals. It has been suggested that whole foods may have 5000–25,000 individual phytochemicals with potentially bioactive properties [4,5]. Cereals and pulses are vital sources of fiber (dietary), minerals, proteins, calories, vitamins, and antioxidants, all of which have been confirmed as functional food constituents [6]. The wide variety of secondary metabolites present in plant sources include glycoalkaloids, antioxidant components, and vitamins, which have been confirmed to have many beneficial effects for human health, for instance antioxidative, anti-inflammatory and cardioprotective properties, in addition to preventing obesity and regulating diabetes [2,7]. Fruit is the fleshy part of plants that is sweet or sour and edible in its raw state. It provides ample sources of sugars, vitamins and bioactive ingredients such as phenols and fiber that have been reported for their biological activity which is related with their ability to attenuate the progression of certain degenerative disorders [8].

Plant-derived natural antioxidants are principally polyphenols, such as stilbenes, anthocyanins, flavonoids, lignans, and phenolic acids, in addition to carotenoids (carotenes and xanthophylls), vitamins C, and vitamin E. These natural antioxidants, in particular carotenoids and polyphenols, possess a wide variety of biological attributes, such as anti-aging, anti-inflammatory, anti-viral, anti-microbial, and anti-cancer properties [9,10,11].

Recently, food-derived proteins or peptides have gained importance due to their bioactive properties, which were shown to be dependent on their amino acid sequences [12]. Bioactive peptides are defined as specific amino acid sequences that exhibit a useful biological activity or with exert a positive influence on human health and body functions, regardless of their nutritive importance [13]. It has been reported that plant proteins contain bioactive peptides (BAPs) as inactive amino acid sequences when present within the native protein. However, BAPs can be readily released by various processes including fermentation, in vitro or human gastrointestinal tract enzyme-catalyzed proteolysis, and food processing [14,15,16]. BAPs have been reported in legumes that may prevent chronic disorders, but research studies related to cereals remain limited [17]. This review focuses on the bioactive components of plant foods, such as fruits, vegetables, cereals, grains, and their potential relevance to human health benefits.

## 2. Research Methods

The search for articles (original or review) was performed using the PubMed, Google Scholar and Scopus electronic databases until October 2020. The main keywords used were: “plant food derived bioactive components”; “gut health modulation”; “oxidative stress and brain health”; “anti-diabetic activity”; “anti-cancerous attributes” and “non-communicable disorders”, among others. All the included references were manually selected and reviewed by the authors.

## 3. Plant-Derived Bioactive Components

Plant-derived bioactive components originating from metabolism are generally known as secondary metabolites, and have promising therapeutic attributes, especially antioxidative properties. Phenolics and carotenoids are considered the key bioactive or phytochemical compounds that can help to maintain better human health [8]. Most of the orange- and yellow-colored fruits and vegetables contain large amounts of lipophilic molecules known as carotenoids [18]. These compounds are very useful for food industrial purposes such as pigments and health-promoting dietary agents. For example, there is evidence indicating that zeaxanthin, Lutein and β-cryptoxanthin have the capability to down-regulate age-linked macular degeneration, protect against disorders related to sunburn, decrease cardiovascular disorders, and prevent cataracts [19,20,21,22]. Furthermore, carotenoids have now attracted much interest due to their proven strong antioxidative activity, which can help to reduce the risk of certain chronic diseases [2].

**Figure 2:**
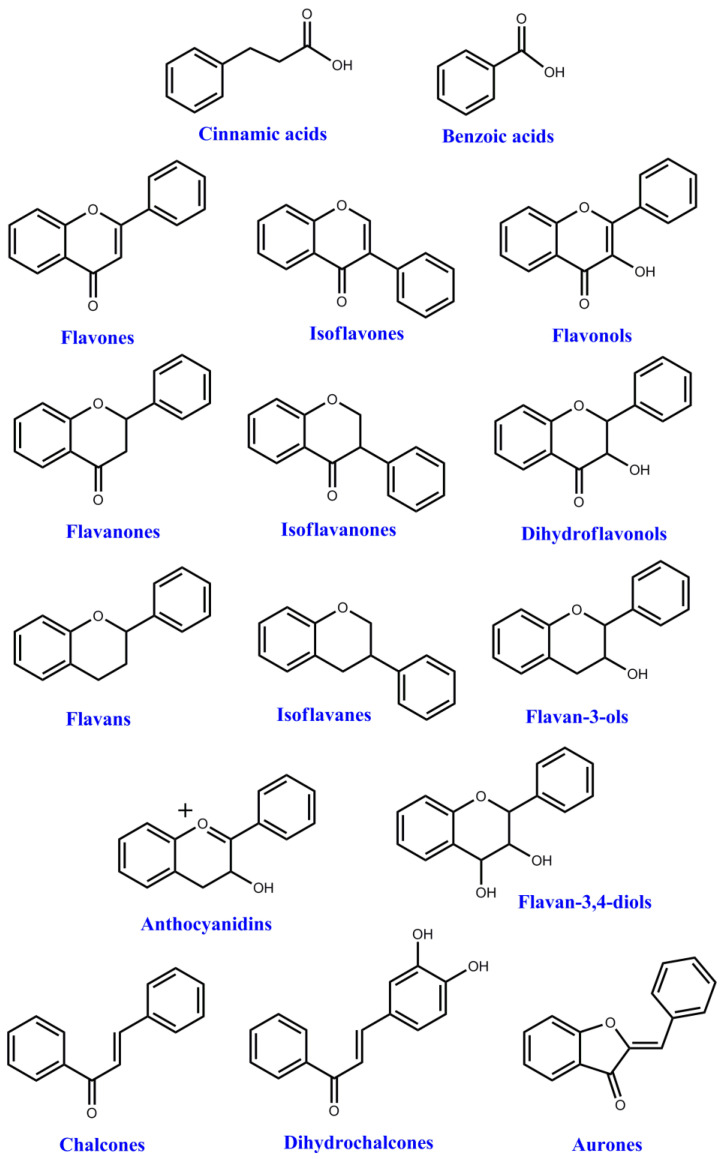
Basic classes of phenolic compounds [5].

Polyphenols are natural antioxidants that are chiefly derived from medicinal plants and food, for instance vegetables, fruits, cereals, medicinal herbs, beverages, spices, and mushrooms. Phenolic acids, flavonoids, and anthocyanins, amongst others, are classes of polyphenols. Natural antioxidants, especially carotenoids and polyphenols, have been reported to possess several biological attributes, such as anti-cancerous, anti-aging, and anti-inflammatory properties [11]. Figure 2 show the structure of polyphenols.

Fruits are rich in hydroxycinnamic acids (HCAs) with values of 0.5–2 g/kg fresh weight in apples, kiwis, cherries, plums, blueberries, etc. Caffeic acid is one of the most plentiful phenolic acids and in certain fruits it consists of about 75%–100% of the total HCAs [23]. However, in cereal grains, ferulic acid is the most abundant phenolic acid and accounts for about 90% of the total polyphenol content of wheat grain [11]. Anthocyanins have been widely used as dyes thanks to their colors, but they can also be useful in regulating neuronal disorders, cancer, cardiovascular diseases, inflammation, diabetes and several other human illnesses. These health-promoting properties of anthocyanins are mainly a result of their strong antioxidative potential [24,25]. Tannins and flavonoids such as epicatechin, catechin and gallocatechin are found in the pulp and soluble cell wall fractions of fruit. Previous findings confirmed that salicylic, p-coumaric, ferulic, p-hydroxybenzoic, vanillic, gentisic, sinapic, syringic, and gallic acids are the main phenolic components in bananas, whereas nitrogen containing biogenic amines are formed by amino acid decarboxylation or aldehyde and ketone amination. Banana pulp and peel have been reported to retain biogenic amines, including norepinephrine, serotonin and dopamine [26,27]. The consumption of vegetables and fruits is recommended as they are a rich source of natural antioxidants, for example vitamin E (tocopherols) and vitamin C (ascorbic acid). Antioxidants have several health promoting attributes including the regulation of immune functioning, reducing DNA damage and ameliorating lipid peroxidation. Vitamin C exists as ascorbic acid and dehydroascorbic acid, with the former being the predominant form in plants [28,29]. Phenolic acids, stilbenes, flavonoids, and lignans are abundant polyphenols in medicinal and food plants. Phenolic acids include cinnamic acid derivatives such as ferulic, p-coumaric and caffeic, as well as benzoic acid derivatives such as hydroxybenzoic acids (HCA) and gallic acid. In edible plants, HCAs are found in higher concentrations than hydroxybenzoic acids [11]. Red wine and tea are the richest sources of dietary catechins. Catechins are typically present in the form of aglycones or esterified with gallic acid. Additionally, luteolin and apigenin are the key flavones, the main sources in the human diet being celery and red pepper. Anthocyanins, including delphinidin, pelargonidin and cyanidin contribute to the violet, red, or blue colors of edible plants such as berries, plums and eggplants. Daidzein and genistein isoflavones are isoflavonoids mainly found in legumes. Soybean and soy-derived products are the richest dietary sources of these compounds [30,31]. Quercetin is a flavonol-type flavonoid widely found in many foods such as apples, onion and tea [32]. Naringenin is a natural flavanone mainly found in grapes and citrus fruits, demonstrating the potential to maintain insulin signaling in the brain and cognitive functioning regulation [33]. Proanthocyanidins, also called condensed tannins, are considered the last member of the flavonoid group. They are catechin (flavan-3-ol) oligomers or polymers that are linked through interflavan bonds. They are found in several varieties of plant foods and beverages such as red wine, grains and fruits [34]. Another, type of plant-derived bioactive is organosulfur compounds (OSCs). Onion (Allium) contains rich amounts of OSCs, and thanks to these biofunctional components, the regular consumption of Allium is associated with the prevention of numerous chronic disorders, such as diabetes, obesity, cardiovascular diseases, and metabolic disorders [35]. Recent evidence reported that major onion thiosulfinate is isoalliin (organosulfur compounds,) together with other precursor compounds, such as S-methylcysteine sulfoxide (methiin), being precursors of a wide range of sensory-active and health-promoting compounds [36].

Previous studies suggested that the regular intake of orange juice (OJ) reduces inflammatory reactions and oxidative stress. OJ flavanones, mainly naringenin-7-rutinoside and hesperetin-7-O-rutinoside, are reported to have a wide range of beneficial attributes on human health such as the improvement of lipid profile [37,38]. Garlic belongs to the Allium genus and is traditionally used as a medicinal plant all over the world. Earlier studies confirmed their health-promoting properties, such as anti-cancer, anti-diabetes, etc. These health benefits are reportedly due to the presence of bioactive compounds such as organosulphur components and phenolic compounds [39]. In the Mediterranean diet, virgin olive oil (VOO) is the best-known element, which is popularized due to its nutritional value and sensory properties. The benefits to health of VOO mainly stem from its phenolic components, for example, hydroxytyrosol, tyrosol, flavonoids, vanillic, and p-coumaric acids. [40].

Plant peptides or protein fragments are usually smaller than 10 kDa. These peptides are present naturally or derived from their native/precursor proteins through enzymatic proteolysis. Bioactive peptides can be obtained from several food sources and can be consumed to influence physiological processes that can be beneficial for human health [41].

## 4. Promising Health Beneficial Attributes of Bioactive Components

Phenolic components are known to be primary antioxidative or free radical inhibitory agents because of their capability to donate hydrogen atoms or electrons [27]. Antioxidative components derived from fruit and vegetables can decrease heart disorders, arthritis, neurodegenerative diseases, cancer, arteriosclerosis, and regulate brain dysfunction [8,29]. Several bioactive peptides isolated from rice, barley, oat, wheat and cereals have been revealed to present antihypertensive activity [41,42]. Protease-assisted food protein hydrolysis can also liberate peptide sequences, which have lipid and cholesterol-lowering actions [43]. Consumption of Allium vegetables such as leek, garlic and onion have been reported to protect against cardiovascular diseases, diabetes and several other metabolic conditions. Furthermore, their intake is linked with the reduction of numerous types of cancers [44]. 

**Figure 3:**
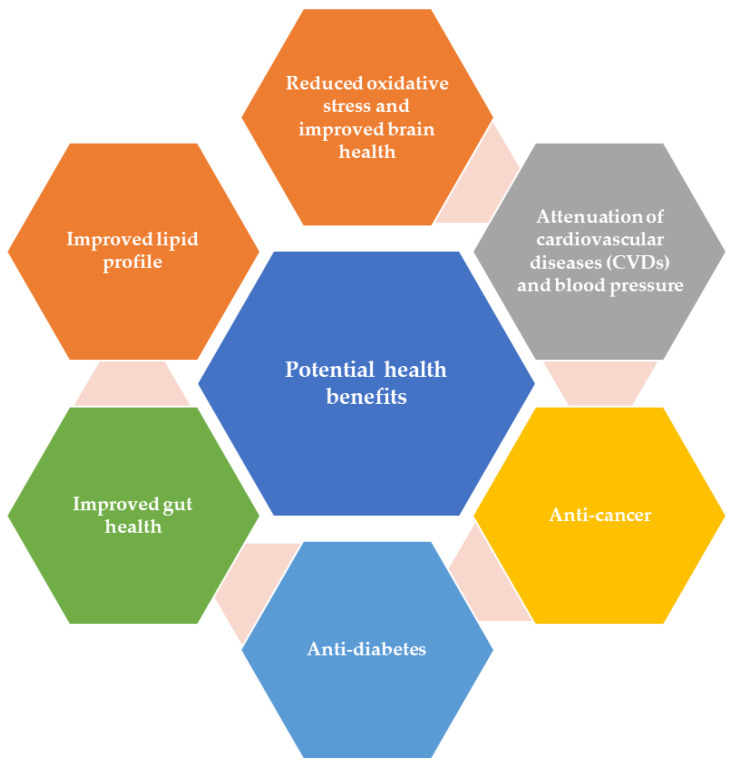
Potential health-promoting properties of bioactive components.

Evidence in the literature shows that plant-derived bioactive compounds have biological attributes such as anti-cancer and antidiabetic activity, the amelioration of cardiovascular diseases, maintenance of oxidoreductive balance and brain health, the improvement of blood lipid profile, and regulation of gut health. Figure 3 shows the promising health-promoting attributes of bioactive components (Graphical abstract).

### 4.1. Attenuation of Cardiovascular Diseases (CVDs) and Blood Pressure

The World Health Organization (WHO) stated that about 17.9 million individuals die of cardiovascular disorders (CVDs) every year worldwide, but that this annual figure will rise to nearly 23 million by 2030 [45]. CVDs are a collection of conditions of the heart and blood vessels including coronary cerebrovascular disorders, heart disorders, rheumatic heart ailment and other conditions [45,46]. For the homeostasis of blood pressure (BP) and sodium (Na), the renin-angiotensin and kallikrein-kinin (KKS) pathways play a vital role [41]. Angiotensin converting enzymes (ACE) transform angiotensin I to angiotensin II and disable bradykinin, a powerful vasodilator which leads to higher blood pressure (BP) and an increased risk of CVDs [47]. Evidence obtained through pre-clinical and clinical studies indicates that a suitable diet can have beneficial on human health including cardioprotective properties. For instance, diets rich in fruit and vegetables are useful to protect against CVDs. Plant-derived bioactive compounds such as peptides, polyphenols, vitamins, oligosaccharides, and fatty acids have cardioprotective properties and can promote heart health [48]. Comprehensive studies have also concluded that plant-derived extracts have the capacity to regulate human BP [49,50]. Data from experimental studies suggest that polyphenols derived from grapes can lower the risk of atherosclerosis through several mechanisms, including reducing low density lipoprotein (LDL) oxidation. Reduced oxidative stress affects the cellular redox state by decreasing inflammation, regulating endothelial functions, preventing cell senescence via activating novel proteins, hindering aggregation platelets, and BP reduction [51]. Furthermore, a pilot study assessed β glucan bioactive effects. The results confirmed that 30 days’ consumption of pasta supplemented with 6% of β-glucan resulted in considerable reduction in the risk of LDL cholesterol and cardiovascular diseases (CVD) [52]. Table 1 shows studies associated with the health benefits of bioactive compounds from plants.

A study by Koyama et al. examined the antihypertensive effect of bioactive peptides from lactic-fermented buckwheat sprouts when orally administered to hypertensive rats. The results showed that the single dose supplementation of six bioactive peptides (DVWY, FQ, FDART, VAE, WTFR and VVG) led to BP reductions in hypertensive rats [53]. In another study, peptide fractions of chia (*Salvia hispanica* L.) seeds were evaluated for the inhibition of ACE activity. The results confirmed that bioactive peptides isolated by Alcalase-Flavourzyme sequential (controlled protein hydrolysis) proteolysis from chia have enormous in vitro ACE-inhibitory properties [54]. Meanwhile, Toufektsian et al. evaluated the cardioprotective effects of anthocyanin-rich maize (20% seed diet) in male Wistar rats after feeding for eight weeks. They found that the rats fed an anthocyanin-rich maize diet had significantly (*p* < 0.01) reduced infarct size, coronary occlusion, and reperfusion than those fed an anthocyanin-free diet, suggesting that anthocyanins may have cardioprotective attributes [55]. Another review study described that hydroxytyrosol, one of the major polyphenols present in olive oil, has the potential to ameliorate heart disease and acts as a cardioprotective agent due to its strong radical-scavenging activities [56].

### 4.2. Anti-Cancer

Epigenetic changes may be produced by altering aspects such as lifestyle and diet. Currently, it could be a crucial step to identify and develop epigenetically based interventions as an anti-cancer approach [90]. Natural dietary components or compounds have the potential to regulate cancer stem cell pathways that are associated to self-renewal, for example the Hedgehog, Notch, and Wnt/β-catenin pathways [91]. Chlorogenic acid (CGA) is a dietary polyphenol that has several important biological capacities. It is produced by several plants and is one of the most representative compounds in coffee. It has been reported to act as an anti-cancer agent by encouraging various human cancer cells linked to apoptosis, for example lung cancer cells and leukemia [92]. A study by Afshari et al. evaluated the anti-cancerous properties of eggplant extract on the human gastric cancer cell line. The authors concluded that eggplant extract had a stronger toxic impact on human gastric cancer cell than the normal cell line. Eggplant has rich contents of phenolic constituents and potent antioxidative properties, which could be effective in the detoxification of free radicals. Consequently, eggplants may be used as a protective food to reduce the incidence of cancer [57]. Another in vitro study assessed the anti-cancer attributes of solasodine, a natural aglycone of eggplant glycoalkaloids. The results showed that solasodine reduces the viability of the A549 cancer cell line and prevents cell invasion (metastasis) by blocking matrix metalloproteinase (MMP) expression. Moreover, solasodine also decreased PI3K/Akt signaling and downregulated the expression of microRNA-21 (miR-21). Overall, the results depict solasodine use as a potentially effective strategy for lung cancer prevention [58]. Plant derivatives such as polyphenols can recover the negative or unhealthy epigenetic mutations in cancer cells, impede tumorigenesis development, prevent metastatic development, or sensitize tumor cells to radio- and chemotherapy [93]. Nevertheless, while diet-based interventions aiming to target epigenetic pathways are definitely promising, the translation of these scientific findings into clinical or public health practices still remains a challenging aspect [90]. A study by Montales et al. evaluated the anti-tumor potential of polyphenols in blueberry (BB) and isoflavone genistein (GEN) in soy. The results concluded that sera from mice ingesting dietary BB repressed the formation of mammosphere in MDA-MB-231 and MCF-7 cell lines. In addition, mammospheres from cells treated with GEN demonstrated significant upregulated expression of PTEN, a tumor suppressor that provokes the PI3K/Akt signaling pathway. Studies of molecular pathways showed that GEN can inhibit AKT, and enhance the expression of PTEN [59]. Another study involving stem-like glioma cells (CD133+) assessed the potential as an anti-cancer agent of Pomiferin (flavonoid extract), a component of the *Maclura pomifera* fruit. It found that Pomiferin impeded the viability and reduced the population of CD133+ cells, in addition to impeding their sphere formation and glioma neurosphere cells invasion capabilities. Moreover, Pomiferin down-regulated genes of glioma cells that are associated with the multiple stemness, such as Nanog, Nestin, and BIM1, during in vitro experiments [60]. A study of the anti-cancer potential of pterostilbene, a blueberry isolate (natural stilbene), showed that it efficiently inhibited the generation of cancer stem cells and their metastatic capacity under the stimulus of M2 TAMs (tumor-associated macrophages) through modifying epithelial-to-mesenchymal transition (EMT) linked signaling pathways, namely the NF-κB/miR488 pathway [61]. Flavonoids from fruit and vegetables are reported to inhibit the NF-κB pathway that is thought to regulate inflammation, angiogenesis, cell proliferation, cell transformation, and the invasion/metastasis and survival of cancer cells. Principally, anthocyanins, flavanones, chalcones, isoflavones, and flavonols, which are derived from legumes, fruits, spices, nuts and vegetables, function as negative regulators of cell signaling pathways that mediate pro-inflammatory responses, promoting the prevention of cancer. They could, therefore, be used as a treatment strategy for cancer [94]. Furthermore, plant-derived carotenoids and terpenoids have been confirmed to possess anti-cancerous and anti-inflammatory attributes. They suppress NF-κB signaling pathways, which are key modulators in the pathogenesis of tumor and inflammatory disorders [95]. A recent ex vivo study by Dobani et al. 2021 evaluated the protective effects of bioactive compounds (polyphenolics) derived from raspberries against colon cancer. The study determined the impact of fermentation on polyphenols in ileal fluid. A significant decrease in polyphenols was found during fermentation, while microbial metabolites significantly increased. Moreover, an oxidative challenge COMET assay showed that post-raspberry ileal fermentate significantly reduced (~30%) DNA damage in the CCD 841 CoN normal cell line. The results indicate that colon-available raspberry polyphenols and their microbial-derived catabolites may play a role in protection against colorectal cancer [96]. Evidence from other research confirmed the anti-cancerous potential of 4 kDa peptide derived from buckwheat. The final results showed that the peptide inhibited the proliferation of HepG2 (hepatoma), liver embryonic WRL 68, breast cancer (MCF-7) and L1210 (leukemia) cells with IC50 values of 33, 37, 25 and 4 µM, respectively [62]. A recent study by Vilcacundo et al. estimated the anti-carcinogenic capacity of quinoa-derived peptides. The findings suggest that LWREGM, DKDYPK, RELGEWGI, DVYSPEAG, and IFQEYI are promising anti-cancer agents due to their chemoprotective and antioxidant activities [63]. Another in vitro study of breast cancer cell lines (MDA-MB-231 and MCF7) investigated the capacity of anthocyanin extracts derived from blueberry and anthocyanin-pyruvic acid adduct extracts as anticancer agents. It confirmed that these extracts show promising anti-cancer capacity in both breast cancer cell lines MDA-MB-231 and MCF7 by hindering cancer cell production and by acting as cell anti-invasive factors [64]. A recent study by Lee et al. confirmed the anti-cancer attributes of sorghum bran extract (high phenolic) on human colon cancer cells. It reported that S-phase growth arrest and apoptosis were stimulated, whereas cell invasion and migration were suppressed through this treatment. These effects were accompanied by the transformed expression of cell cycle, apoptosis and metastasis adaptable genes, using sorghum bran extract prepared with 70% ethanol in 5% citric acid to treat cancer cells [65]. Another recent study by Henidi et al. confirmed the protective properties of quercetin against vascular toxicity induced by doxorubicin and its influence on the therapeutic cytotoxic profile of doxorubicin (anticancer agent) in cancer cell lines (breast). Quercetin combined with doxorubicin were found to double the IC50 of doxorubicin alone in MCF-7 cells from 0.4 ± 0.03 to 0.8 ± 0.06 μM. Furthermore, a staining technique (annexin-V/FITC) showed that the combination of doxorubicin/quercetin resulted in considerably fewer apoptotic cells compared to the cells treated only with doxorubicin. The study concluded that quercetin, in addition to its effective vascular protective ability compared with doxorubicin, affects doxorubicin-induced anti-breast cancer potential through cellular pharmacokinetic and pharmacodynamic characteristics [97].

### 4.3. Anti-Diabetes

Diabetes is a metabolic disease that results in several conditions or symptoms such as glucose intolerance, elongated hyperglycemic condition, disturbed metabolic energy utilization and storage [98]. The disease also includes the compromised anabolism and catabolism of lipids, carbohydrates and proteins due to deficient insulin production and improper functioning of the insulin receptor. It is a disease that is harmful to health and is becoming more prevalent worldwide. The WHO reported that the number of diabetes mellitus (DM) patients has reached 415 million globally [99]. A research study by Cheng et al. reported that Rutgers Scarlet Lettuce (RSL) polyphenol-rich aqueous extract could be used as a potential anti-diabetic agent. RSL extract (RSLE) at the dose 100 or 300 mg/kg was found to improve the glucose metabolism in high-fat diet induced obese mice after 28 days of oral administration. Moreover, in vitro treatment of H4IIE rat hepatoma cells with chlorogenic acid and RSLE suggested the dose-dependent prevention of glucose production. Overall, the results suggested that RSLE had anti-diabetic attributes during in vivo and in vitro trials [66]. Other research activities reported the potential of quinoa-derived bioactive peptide fractions as anti-diabetic agents. In vitro findings confirmed that three bioactive peptide fractions from 11S seed storage globulin B showed inhibitory activities against α-glucosidase, α-amylase, and dipeptidyl peptidase IV (DPP-IV) enzymes [67]. Another study investigated the possible health attributes of Chungkookjang (short-term fermented soybeans with *bacillus lichemiformis*) in relation to anti-diabetic efficacy. Chungkookjang was shown to enhance insulinotropic functioning in islets of type 2 diabetic rats, which was endorsed due to the existence of isoflavonoid aglycones and small peptides. Moreover, it was suggested that small peptides (<3 kDa) improved insulinotropic functioning via PPAR-γ antagonist action [68]. A study by Li et al. estimated the anti-diabetic attributes of anthocyanin using fifty-eight diabetic patients in a 24-week clinical trial. The diabetes group consumed two anthocyanin capsules (160 mg) purified from bilberry and blackcurrant twice/day. The group that consumed anthocyanin capsules had a substantially lower insulin resistance index and fasting plasma glucose, along with considerably raised serum β-hydroxybutyrate and adiponectin concentrations in comparison to the placebo group [69]. A study by Zhang et al. confirmed the anti-diabetic potential of oat-derived peptides in streptozotocin-induced diabetic mice. The oat seed protein hydrolysate obtained from alkalase digestion provided bioactive peptides that inhibited the α-glucosidase enzyme. Furthermore, the oat peptides had a hypoglycemic action in STZ-induced diabetic model of mice, enhanced secretion of insulin, decreased food intake, promoted glycogenesis and increased insulin sensitivity [70]. Walnut hydrolyzed peptides (WHPs) from proteins of *Juglans mandshurica* fruits exhibited anti-diabetic attributes by enzyme inhibitory action. An in vitro study of WHPs indicated that 3–10 kDa peptides reduced α-glucosidase activity and improved extracellular glucose consumption in HepG2 insulin-resistant cells. During in vivo tests, the WHPs decreased fasting blood glucose levels (64.82%) by improving liver glucokinase, glycogen and insulin secretion by 69.54%, 76.19%, and 23.71%, respectively [71]. A recent study by Barik et al. confirmed the potential of anthocyanin-rich black currants (BC) to regulate hyperglycemia using CaCo-2 cells. Results concluded that anthocyanins in BC control the postprandial hyperglycemia primarily by impeding α-glucosidase activity, while other phenolics regulate sugar transporters, glucose uptake and salivary α-amylase, which together could reduce the linked risk of evolving type-2 diabetes [72]. Yang et al. conducted research to investigate the glucose-lowering potential of puerarin (isoflavones) using STZ-induced diabetic mouse models. They found that, after four weeks of treatment, the puerarin group showed a significant reduction in blood glucose (hypoglycemic effects) and an enhanced blood insulin level. Furthermore, oral glucose tolerance tests (OGTT) and destroyed islets cells were improved in the group administered puerarin. This isoflavone has also been found to upregulate p-AKT and p-GSK-3β levels in liver tissue and, in contrast, to downregulate the mRNA level of uncoupling protein (UCP2). Overall, the study proposed that puerarin could improve hyperglycemia in STZ mice by protecting pancreatic β-cells and enhancing liver functions through the activation of AKT signaling and down-regulation of UCP2 expression [100].

### 4.4. Gut Health

Evidence from epidemiological and animal studies suggests that “gut health” can be considered as a novel research objective in medicine. It is not only a target for the treatment of extensive gastrointestinal conditions, but has attracted interest as a suitable approach for the protection of human health and resistance to illnesses [101]. The epithelial barrier of the gut is a multifaceted construction that divides the host gut lumen and the internal environment. The gut barrier is not only composed of mucus layers and epithelial cells, but also of several other components, such as the immune system (e.g., cytokines, immunoglobulin A), microbiome, and intestinal vascular, neurenteric, and endocrine systems [102,103]. Lipids and cholesterol metabolism could be influenced by gut microbiome through controlling the particular metabolic signaling pathways attributed to the bile acids properties, which functions as signaling molecules. Through binding to various cellular receptors, bile acids could perform actions, e.g., G-protein-coupled receptors TGR5 and farnesoid X receptor (FXR). Primary bile acids stimulated the FXR signaling, which participates in the regulation of several target genes associated to lipid and carbohydrate metabolism, and innate immunity of the gut [104].

Plant-based foods, such as fruit and vegetables, are rich sources of carbohydrates and fiber and should be consumed as the bulk of the human diet. They also contain large amounts of other compounds (phytochemicals) that have additional impacts on the gut microbiome [105]. Apart from polyphenolic compounds, it has been suggested that modifications of gene expression by protein hydrolysates and essential amino acid sequences (peptides) could produce a hypocholesterolemic effect through binding neutral sterols and bile acids in the gut and lead to the removal of fecal content [43]. A recent clinical study of 51 adults by Hidalgo-Liberona et al. confirmed the potential of polyphenols to modulate gut health and prevent unhealthy/pathological situations that are associated with distorted intestinal permeability (IP). The results suggest that reduced polyphenol bioavailability due to IP may be attributed to disturbances in the phase II methylation processes and metabolism of intestinal microbes. Moreover, it was determined that metabolites derived from microbiota may possibly be liable for the biological action provoked by polyphenols in the age-linked disturbance of IP [103]. Through human fecal bacterial batch-culture fermentation, a previous study revealed that (−)-epigallocatechin, (+)-gallocatechin gallate, and (−)-Epigallocatechin gallate from oolong tea promoted the expansion of the useful *Bifidobacterium* spp. and *Lactobacillus*/*Enterococcus* group, while preventing the development of harmful *Eubacterium*-*Clostridium*, *C. histolyticum*, and *Bacteroides–Prevotella* groups [73]. The study concluded that oolong tea extracts have the potential to modulate intestinal microbiota and confer health benefits to the host. In a fourteen-month dietary interventional study, extract of grape pomace (rich in phenolic compounds) affected gut microbiota modulation in rats. The study concluded that the grape pomace extract enhanced the beneficial *bifidobacteria* population, while in the control subjects, grape pomace consumption reduced the abundance of *Clostridium* sensu stricto (cluster I) [74]. Another study evaluated the potential of proanthocyanidins derived from grape seed to modulate gut microbiota and intestinal barrier integrity by means of a weaned piglet model [75]. It was concluded that the piglets given proanthocyanidins presented enhanced growth performance, significantly decreased diarrheal occurrence, reduced intestinal permeability, and enriched mucosal morphology. Furthermore, proanthocyanidins improved the antioxidant indices in intestinal mucosa and serum, while also enhancing occludin (intestinal barrier gene) expression. In addition, proanthocyanidins increased the amount of Clostridiaceae and decreased the abundance of Lactobacillaceae, suggesting that dietary proanthocyanidins enriched the diversity of microorganisms in the gut.

An earlier study by Anhê, et al. determined that a polyphenol-rich extract of cranberry improves intestinal inflammation in mice. The findings indicated that eight weeks supplementation of a polyphenol-rich cranberry extract significantly enhanced the Akkermansia spp. population, which was related to declines in gaining weight, intestinal inflammation, and visceral obesity, increased by a high-fat/high-sucrose diet [76]. In a recent review, Gong et al. reported the health benefits of whole-cereal grains, in relation to the modulation of gut microbiota due to their rich phenolic content and dietary fibers. It has been suggested that cereal rich foods with suitable processing techniques could be used for the improvement and even management of several metabolic illnesses [106]. Polyphenolic compounds and dietary fibre exist in foods and could be utilized by bacteria to form metabolites such as phenolic acids and short-chain fatty acids (SCFA) that can promote human health. Hence, the dietary fibre and phenolic content of foods could play a beneficial part in maintenance of gut microbiota arbitrated management as well as prevention of chronic disorders. Disturbance of gut microbiota is also known as gut dysbiosis that is linked to several health aliments in the host [104]. Moreover, SCFAs transported in the blood circulation function as vital controlling molecules in the mitogen activated protein kinases (MAPK), AMP-activated protein kinase, and peroxisome proliferator-activated receptor gamma (PPARγ) signaling pathways to facilitate lipid biosynthesis and gluconeogenesis [106]. A recent research study by Nagata et al. has stated that the combination of adzuki bean (polyphenol rich) extract and inulin enhanced the concentration of propionic acid and reduced the level of ammonia-nitrogen in the caecum of rats. The results confirmed that phenolic compounds/extracts and dietary fiber could be used to make food ingredients in functional food that can regulate gut health [77]. Kim et al. assessed the potential of *Porphyra tenera* (PT) seaweed food in relation to gut microbiota modulation or gut health of mice fed with DSS plus PT extracts, dextran sodium sulfate (DSS)-fed mice, and control mice. The authors found that PT extract considerably altered the gut microbiota of mice, improved the abundance of genera *Clostridium_XIVb* and also enhanced some of the expected metabolic events. They concluded that PT extract could improve colitis inflammation induced by DSS via modifying the gut microbiota functions and compositions in mice [78]. Metabolites that are produced from cereals and other plant food diets through the gut microbiome, e.g., secondary bile acid, phenolic metabolites, and SCFAs, as a result encourage host physiology through several mechanisms. Microbial metabolites are deliberated to be the utmost significant regulators of the human metabolism. They could also control energy and appetite homeostasis, metabolism of glucose and lipids, in addition to modifying oxidative stress and inflammation via diverse signaling pathways [106].

### 4.5. Lipids Profile Regulation

Published scientific evidence has suggested that bioactive compounds have the capacity to maintain lipid homeostasis. Flavonoids and phenolic components have important properties that can regulate lipid equilibrium [107,108]. Anthocyanins extracted from eggplant peels were investigated for their antihyperlipidemic activity in rats. The study claimed that anthocyanins from eggplant peel significantly reduced the levels of very low-density lipoprotein (VLDL), low-density lipoprotein cholesterol (LDL-C), serum total cholesterol, and triglyceride in rat model, whereas the high-density lipoprotein cholesterol (HDL-C) were significantly improved [79]. It has been argued that adding eggplant-derived anthocyanins to the diet can reduce obesity by reducing serum cholesterol and triglycerides and improving concentrations of high-density lipoprotein cholesterol [2]. Natural plant sterols such as phytosterols are used as functional food ingredients due to their extensive range of potential attributes for promoting health, such as blood cholesterol reducing activity and decreasing intestinal absorption of cholesterol. Due to their structural resemblance with cholesterol, these components prevent cholesterol solubilization in the intestine, which leads to decreased absorption. Moreover, they can compete with low-density lipoprotein (LDL) cholesterol to cause a reduction in low-density lipoprotein cholesterol levels inside blood vessels [27,109]. Studies in the literature have confirmed that the daily consumption of up to 3 g/day phytosterols is safe and has a hypocholesterolemic action in patients requiring a noticeable decrease in low density lipoprotein (LDL) cholesterol levels in plasma [110]. It has been stated that flavonoids can enhance the elimination and degradation of cholesterol through neutral sterols and bile acids. Oral administration of flavonoids extracted from unripe fruits of *Musa paradisiaca* at a dose of 1 mg/100 g body weight (BW) showed significant hypolipidemic activities in male Sprague Dawley rats. Moreover, substantial increases in the hepatic and fecal neutral sterols and bile acids were also detected, which demonstrated a greater degradation rate of cholesterol [80]. Galvez has been granted a patent for lunasin biological activity in relation to cholesterol reduction. The patent provides a method for cholesterol level reduction and the maintenance of an individual’s overall lipid profile by reducing total cholesterol and LDL cholesterol by consuming effective amounts of lunasin [111]. Fortified bread with *Moringa oleifera* (MO) leaf powder demonstrated its potential as anti-hyperlipidemic agent using a rat model [81]. The results confirmed that breads fortified with MO at 10% and 15% were the best for ensuring decreases in total cholesterol, triglycerides, low density lipoprotein and very low-density lipoprotein. MO leaves also contain β-sitosterol, a bioactive compound with known cholesterol-lowering properties that may have been responsible for the reduction of plasma cholesterol levels in rats fed a high fat diet. Neyrinck et al. confirmed that the administration of a polyphenol-rich pomegranate peel extract can reduce the high cholesterol level associated with a high-fat diet in mice. They concluded that the extract of pomegranate peel improved the expression of inflammatory markers associated with a high fat diet in the visceral adipose tissue and colon of mice [82]. A recent study by Li et al. confirmed the lipid lowering capacity of *Idesia polycarpa* (edible oil plant) defatted fruit residue. It was concluded that the polyphenol contained in the fraction D of ethyl acetate extract (EF-D) was responsible for its strong in vitro antioxidant ability. Moreover, EF-D showed a considerable lipid reducing effect on hepatic steatosis in HepG2 cells induced by oleic acid through regulating the expression of genes that are linked to inflammation and lipid metabolism, leading to increased antioxidant activity and decreased liver damage [83]. Xu et al. examined the effect of phospholipids, tocopherols, sterols, coenzymes CoQ10 and (Co) Q9 and phenols on antioxidant defenses and plasma lipids. Rats’ liver and plasma samples were assessed after being fed a diet of 20% fat and rapeseed oil. The authors reported decreased cholesterol and triglycerides levels, which depended on the administration of the micronutrients [112].

### 4.6. Oxidative Stress and Brain Health

To lead a healthy life, it is necessary for the metabolic processes to occur smoothly. However, these processes release unhealthy entities that are known as reactive oxygen species (ROS). The primary source of ROS is mitochondrial electron transport chain processes that are liberated during incomplete reactions involving oxygen [2]. Carotenoids have been reported to promote good health thanks to their exceptional physiological efficacy as pro-vitamins and their potential by way of antioxidative reactions, particularly in scavenging singlet oxygen. Moreover, they can reduce disease risks, specifically certain types of cancers that are now a growing problem worldwide [27]. A recent article reported that bioactive molecules such as phytosterols and terpenes have the potential to ameliorate neuroinflammation and other neurodegenerative illnesses [113]. Furthermore, there is evidence that grape juice and other fruit and vegetable extracts are immense sources of antioxidants, which can decrease the impact of oxidative stress associated with aging, resulting in improved brain functions [114]. Lu et al. confirmed the promising attributes of the phytoestrogen secoisolariciresinol diglucoside (SDG) with regard to the health of *Caenorhabditis elegans.* The authors found that SDG can prolong the lifespan of *C. elegans* by up to 22.0%, decrease the lethality of oxidative stress and heat, reduce body movement decline linked to age, assuage dopamine nervous deterioration encouraged by 6 hydroxydopamine (6-OHDA), and reduce the toxicity of Aβ protein in *C. elegans* [115]. Resveratrol, a polyphenol in red wine, is a well-researched biomolecule that is reported to improve brain health by acting as an inhibitor of beta amyloid protein aggregation [116,117]. A recent study evaluated the effects of isothiocyanate isolated from *Moringa oleifera* (MO) on both a Parkinson’s disease (PD) mouse model and in RAW 264.7 macrophages stimulated with LPS. The results confirmed the pronounced efficacy of the MO bioactive compound, which results from myrosinase hydrolysis positively modifying oxidative stress and inflammatory and apoptotic pathways [84]. Nascimento et al. identified the potential benefits of *Ganoderma lucidum* mushroom in ameliorating the brain health of rats which consumed ethanol (3 g/kg/day). The results confirmed that the aqueous extract of *G. lucidum* (AEGl) stimulated the recovery of rats’ anxiogenic- and depressive profile, spontaneous horizontal exploration capacity, along with the short-term memory impairment associated with binge ethanol exposure. Consequently, AEGl can lead to positive behavioral changes in a model of psychiatric, cognitive, and motor complaints, and can be regarded as a beneficial food complement tool in the management of illnesses that arises from excessive alcohol consumption [85]. A recent study by Wattanathorn et al. concluded that a phytosome consisting of a combined mulberry and ginger extract (PMG) has the potential to mitigate dementia and memory damage after an ischemic stroke in Wistar rats. The authors found that PMG reduced acetylcholinesterase, interleukin-6 (IL-6), and malondialdehyde (MDA), but increased glutathione peroxidase (GSH-Px), superoxide dismutase (SOD), catalase (CAT), neuron density, and signal transduction through the extracellular signal-regulated kinase (ERK) pathway. Overall, PMG was found to significantly improve memory [86]. During oxidative stress, the ROS formed in the cells can damage lipids, proteins and nucleic acid. Banana is said to be a rich source of antioxidative bioactive components that can protect the host body from several ailments during oxidative stress [118]. An in vitro study assessed whether flavonoids in lemon seeds (FLS) were capable of improving the peroxide-induced oxidative damage of human embryonic kidney 293T cells (HEK 293T cells). Their results showed that the survival rate of the damaged cells improved by up to 76.2% after treatment with 150 μg/mL of flavonoids, and peroxide-induced apoptosis was inhibited. Furthermore, Western blot analysis and quantitative polymerase chain reactions confirmed that FLS enhanced the protein and mRNA expressions of GSH1, SOD1, SOD2, CAT, and GSH-Px in HEK 293T cells in which oxidative damage was induced by H_2_O_2_. The high-performance liquid chromatography investigation established that FLS contained hesperidin, epicatechin, caffeic acid, vitexin, quercetin, and gallocatechin [87]. Citrus fruits and their juices are the main dietary source of flavanones. There is evidence that isoprostane, an oxidative stress marker, decreased in triathletes administered a flavanone-rich aronia-citrus juice for 21 weeks [119]. A recent study by Quesada-Gómez et al. described how β-Cryptoxanthin (carotenoid) inhibits angiogenesis via retinoic acid receptors. Thus, carotenoid-rich foods may be helpful for the treatment and regulation of angiogenic pathological conditions, including wet macular degeneration and cancer growth linked with aging [120].

## 5. Limitations/Risks

The above-mentioned studies confirmed the promising benefits to human health of plant-derived components via studies mainly involving in vitro or animals’ models. However, very few clinical studies have confirmed their health-promoting potential. Therefore, it is necessary to conduct long-term extensive human studies to establish the potential of these bioactive components, prior to recommending their use in human health applications.

## 6. Concluding Remarks

The present study of the literature suggests that plant-derived bioactive compounds have attracted a great deal of consumer interest in relation to improving human health. Several research studies have confirmed the potentially promising attributes of plant-derived bioactive constituents such as biologically active proteins, polyphenols, phytosterols, biogenic amines, carotenoids, organosulfur compounds, which can reduce the severity of several chronic disorders. This paper reviews the scientific evidence relating to plant-derived bioactive ingredients with regard to the regulation, prevention, or treatment of cancer, diabetes, CVDs, blood pressure, oxidative stress, and brain health, gut health, and lipid profile. This research field is now gaining much importance with the discovery of new molecular targets for various human diseases. However, only a limited number of studies have used human intervention trials to confirm the potentially promising attributes of plant-derived bioactive constituents. Therefore, future research activities that involve human trials are required to ensure a greater impact of functional foods as acceptable therapeutic agents.

## 7. Future Prospects

**Figure 4:**
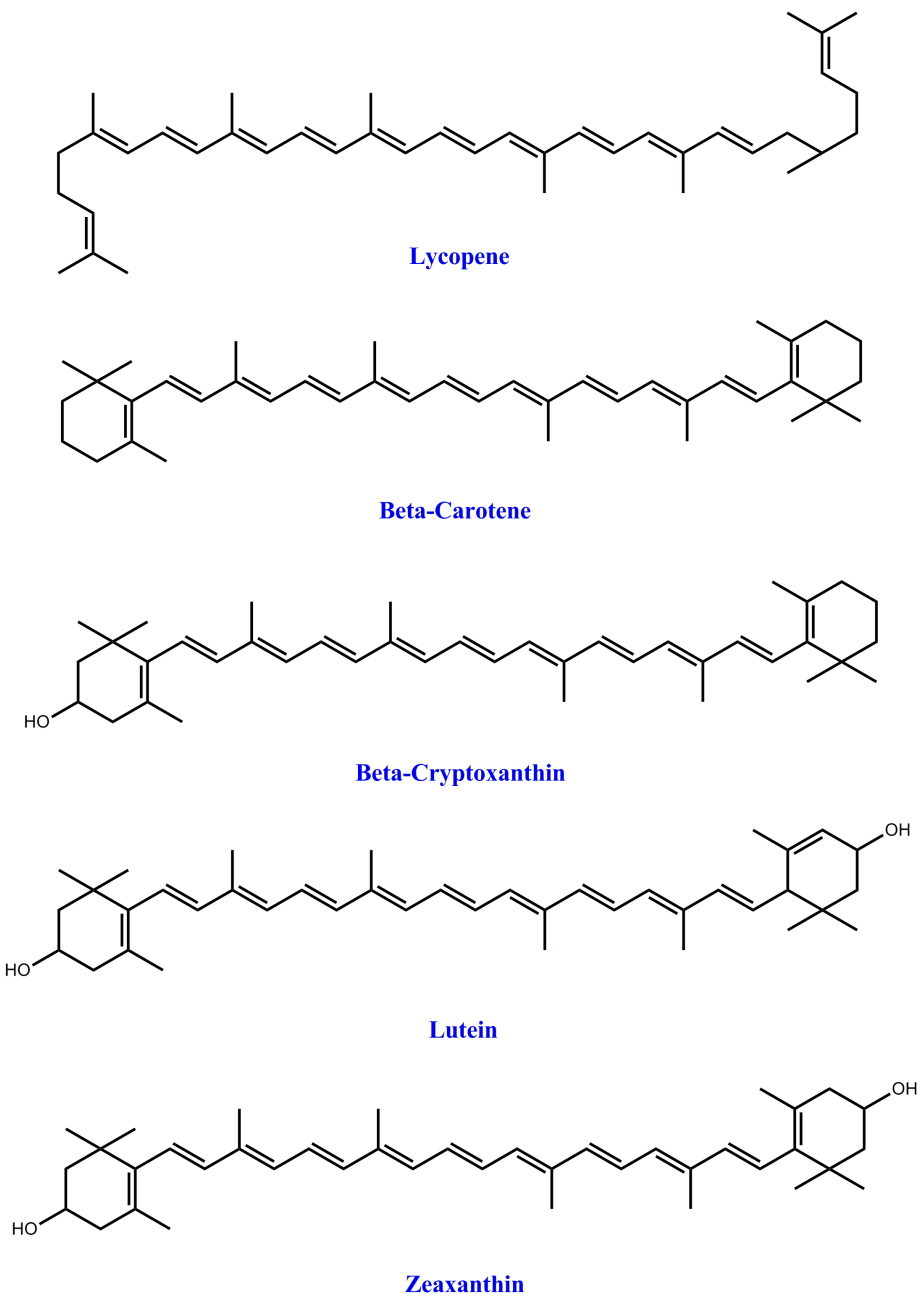
Structures of the most abundant carotenoids [122].

Plant diets contain a multifarious collection of bioactive components, such as polyphenols terpenes, limonoids, carotenoids, each displaying different biological attributes. Fruit, food grains, vegetables, and millet are claimed to have beneficial physiological properties resulting from the large amounts of functional or bioactive components they contain and their nutritive value [6]. Dietary food supplements are consumed to fulfill necessary dietary needs. They are not proposed to diagnose, treat, prevent, mitigate, or cure ailments. In addition to other the polyphenolic compounds, these bioactive food components include vitamins, fiber, minerals, fatty acids, and amino acids. It is assumed that about 50,000 dietary components exist, the majority of which are various multivitamins. These are taken in a therapeutic form, that is via a powder, tablet, or pill, but not in the formulation of conventional foods or beverages (such as juices, soups, etc.) [51]. Leucoanthocyanidin lycopenes, carotenoids, betalains, and anthocyanins are the main phenolic compounds the give fruits and vegetables their color. They are potent antioxidants and may have therapeutic attributes, making them attractive as “functional foods” for betterment of human health. Fruit and vegetables have a vital role in our diet and lives, and hence the claims for such important food commodities have exponentially increased because of the growing world population and the changes in eating habits [121]. Figure 4 show the structure of carotenoids.

Plant-derived components are considered less toxic and are currently used for cancer treatment worldwide, which is accepted by patients, but this trend should be carefully reviewed since not all natural compounds are beneficial [123]. Furthermore, oncology treatment has involved the widespread use of herbal medicines such as *Taxus baccata, Taxus brevifolia* Nutt., *Podophyllum peltatum* L., and *Catharanthus roseus* as complementary therapy. As a result, every year, several new cytotoxic components are isolated to establish new potential compounds for cancer therapy [124]. In this sense, it may be important to understand and divulge how improved eating habits can make our lives healthier and can play a significant role in the management and prevention of non-communicable disorders. Diets rich in polyphenols would be helpful, and the regular consumption of fruit and vegetables as a healthy habit may be useful to reduce the risk of chronic degenerative diseases and metabolic syndromes [125]. In this regard, there is a real necessity for comprehensive research into the application of traditionally used plants as reservoirs of bioactive ingredients. Additionally, before products are prepared, meticulous research and clinical trials must be performed to confirm low dose efficiency that can provide information on the mechanism of action inside the human body [6].

## Figures and Tables

**Table 1:** Promising health benefits of plant originated bioactive compounds.

Plant Food	Bioactive Components	Treatment	Health Benefits	References
**Buckwheat**	Bioactive peptides	Single oral dose of 0.010 mg/kg BW administered in spontaneously hypertensive rats; blood pressure was recorded at 0, 3, 6, 9, and 24 h after administration.	Lowering of blood pressure	[53]
**Chia seeds**	Bioactive peptides	Measurement of ACE-I inhibitory activity through regression analysis of ACE-I inhibitory activity (%) vs. peptide concentration, and IC50 values (in vitro enzymatic activity)	ACE-inhibitory activity (Blood pressure)	[54]
**Maize seed**	Anthocyanins	8 weeks anthocyanin-free (ACN-free) or anthocyanin-rich (ACN-rich) diets were fed to male Wistar rats	Reduction of cardiovascular disease	[55]
**Olive oil**	Polyphenols	Randomized 50 participants ingested 60 mL/day (polyphenol olive oil (360 mg/kg polyphenols))	Reduction of cardiovascular disease	[56]
**Eggplant**	Eggplant extract	Using different concentrations of 10, 5, 2.5, 1.25, 0.6, 0.3, 0.15, 0.06, 0.03, 0.01 mg/mL MTT test was performed using cells for 48 h.	Toxic effect on cancer cells	[57]
**Eggplant**	Glycoalkaloids (solasodine)	Cells (A549) were seeded in a 96-well plate and treated with solasodine in triplicate	Anti-cancer activity	[58]
**Soy**	Isoflavone genistein	Genistein concentration range (40 nM and 2 μM) were tested using MDA-MB-231 and MCF-7	Anti-cancer activity	[59]
***Maclura pomifera***	Pomiferin (flavonoid extracts)	Using the MTS assay (1 and 10 μM); inhibits the both U373 and U87 neurosphere cells growth	Anti-cancer activity	[60]
**Blueberries**	Pterostilbene	Pterostilbene concentration range (2.5, 5, and 10 μM) was added 6 h after seeding MDA-MB-231 or MCF7 cells; MDA-MB-231 cells were cocultured with M2 TAM and subcutaneously injected into the NOD/SCID mice), pterostilbene-treated and control groups (5 mg/kg, ip injection, 5 times/week).	Anti-cancer activity	[61]
**Buckwheat**	Bioactive peptide (4 kDa peptide)	Cancerous cell lines L1210, HepG2, MCF 7, and WRL68 were seeded into each well of a 96-well culture plate (treated with various concentration)	Anti-cancer activity	[62]
**Quinoa**	Bioactive peptides	Caco-2, HCT-116 and HT-29 cells were seeded in 96-well Plates; quinoa protein concentrate digests, fractions or blanks at different concentrations (4 to 0.031 mg/mL) for 24 h	Anti-cancer activity	[63]
**Blueberry**	Anthocyanins	MDAMB-231 and MCF-7 cells were used; anthocyanin-pyruvic acid adduct extract and blueberry anthocyanin extract at 250 μg/mL, for 24 h	Anti-cancer activity	[64]
**Sorghum**	Phenolic (sorghum bran extract)	Human colon cancer cells were cultured in Dulbecco’s modified Eagle medium; cells treated with extract of phenolic sorghum bran having concentration 0, 0.625, 1.25, 2.5, 5.0, and 10.0 mg/mL. (diluted 70% ethanol + 5% citric acid)	Anti-cancer activity	[65]
**Lettuce**	High polyphenol content	For 28 days vehicle (water), Metformin (250 mg/kg) or Rutgers Scarlet Lettuce extract (100 or 300 mg/kg) were orally administered to obese mice (high fat diet-induced)	Anti-diabetic effects	[66]
**Quinoa**	Bioactive peptides	a-amylase inhibition assay, negative control (distilled water) or positive control (2 mM acarbose), 50 µL of sample, added to 100 µL a-amylase solution; a-glucosidase inhibition assay, 100 mL of sample, negative control (distilled water) or positive control (1 mM acarbose) were added to 50 mL of rat intestine a-glucosidase	Anti-diabetic effects	[67]
**Soybean**	Isoflavonoid aglycones and small peptides	Type 2 diabetic rats were administered 10% cooked soyabeamns, 10% traditional chungkookjang, or standardized chungkookjang fermented with *Bacillus lichemiformis* (for 8 weeks)	Improved insulinotropic activity	[68]
**Bilberry and blackcurrant**	Anthocyanin	For 24 weeks 58 diabetic patients were fed with 160 mg of anthocyanins two times/day or placebo (*n* = 29/group)	Anti-diabetic effects	[69]
**Oat seed**	Bioactive peptides (proteins hydrolysate)	For four weeks diabetic mice groups were orally supplemented with high oat peptide, medium oat peptide and low oat peptide 1.0, 0.5 and 0.25 g/kg body weight in the solutions containing 0.6, 0.3 and 0.15 g mL^−1^ oat peptides solution, respectively.	Anti-diabetic effects (inhibits alpha-glucosidase enzyme)	[70]
***Juglans mandshurica*** **(Manchurian walnut)**	Walnut hydrolyzed peptides	Diabetic mice administered Met hydrochloride 125 mg/kg/day; WHPs (3–10 kDa molecular weight) 200 mg/kg/day; WHPs (3–10 kDa molecular weight) 500 mg/kg/day; and WHPs (3–10 kDa molecular weight) 800 mg/kg/day (for 4 weeks)	Anti-diabetic activity	[71]
**Black currants**	Anthocyanins	α-amylase, α-glucosidase assay (in vitro) using 96-well plate format	Anti-diabetic activity (type-2 diabetes)	[72]
**Oolong tea**	(−)-Epigallocatechin gallate, (+)-gallocatechin gallate, and (−)-epigallocatechin	EGCG, GCG and EGCG3”Me on human intestinal microbiota in vitro were evaluated by monitoring bacterial populations; Fermentation was initiated by adding 150 μL of fecal slurry to 1350 μL of culture medium containing EGCG, GCG, EGCG3”Me or FOS (positive control)	Gut health (promoted the growth of beneficial bacteria)	[73]
***Grape***	Grape pomace extract (phenolic compounds-PC)	For 14 months, diets of four groups (2 months old rats) were supplemented with various PC concentrations (diluted in 0.1% DMSO), 2.5, 5, 10, and 20 mg/kg/d) and 1 group administered 0.1% Dimethyl Sulfoxide (DMSO) alone (control group)	Gut health	[74]
**Grape**	Proanthocyanidins	For 28 days, four groups of weaned piglets (28 days) were fed a control diet, or administered with 250 mg/kg proanthocyanidins and half-dose antibiotics or 250 mg/kg proanthocyanidins, kitasamycin/colistin.	Gut health	[75]
**Cranberry**	Polyphenols	For 8 weeks, high fat high sugar mice were orally intubated with cranberry extract (CE) (200 mg/kg) or vehicle (water)	Gut health	[76]
**Adzuki bean**	Polyphenols	Through in vitro (48 h) and in vivo (rats) 28 day study, effects of inulin (INU) and polyphenol-containing adzuki bean extract (AE) on intestinal fermentation were evaluated; final concentrations of pig feces, AE, nitrogen source and carbohydrates in the fermenters were 2, 1, 0.8, and 3% (*w*/*v*), respectively; experimental diets based on the AIN-93G diet (INU + 4% AE; INU + 1% AE; INU; CEL + 4% AE; CEL + 1% AE; and CEL), CEL-High cellulose	Gut health	[77]
***Porphyra tenera*** **(PT) seaweed food**	*Porphyra tenera* (PT) extract	Colitis-induced mice model was used; for 1 week DSS was administered using drinking water, and in mice gastrointestinal tract PT extract was ingested.	Gut health	[78]
**Eggplant**	Anthocyanins	8 week study; six groups of rats were formed and one group was fed a standard pellet diet, other groups fed with high-fat diet; 3 groups (3–5) given 200, 400 and 800 ppm of eggplant peel anthocyanins and group 6 received tertiary butylhydroquinone (TBHQ) (200 ppm).	Reduction of cholesterol levels	[79]
***M. paradisiaca***	Flavonoids	Male rats were administered extracted flavonoids at a dose of 1 mg/100 g (BW)/day	Reduction of cholesterol levels (hypolipidemic activities)	[80]
***Moringa Oleifera***	Leaves powder	Rats were fed for 45 days; Group 1: basal diet; Group 2: (positive control) basal diet, in addition 2% cholesterol to induce (hyperlipidemia); Group 3 and group 4: same composition as positive diet, in addition fortified bread with 10% & 15% MO leaves powder	Cholesterol lowering effects	[81]
**Pomegranate**	Pomegranate peel extract	4 weeks study period; male Balb/c mice were divided into three groups: a control (CT) group, one fed with HF diet and other group fed the same HF diet and also given pomegranate peel extract at a dose of 0·2% (6 mg/d per mouse).	Reduce serum cholesterol level	[82]
***Idesia polycarpa*** **(edible oil plant)**	Polyphenols	TGs were measured using an enzymatic method kit using HepG2 cell	Lipid-lowering effect	[83]
***Moringa Oleifera***	Isothyocynate	For 1 week mice were pretreated with moringin (10 mg/kg + 5 μL myrosinase/mouse/day) and with GMG (10 mg/kg).	Improved oxidative stress and brain health	[84]
***Ganoderma lucidum***	Aqueous Extract of *G. lucidum* (AEGI)	Beginning at 35 days old, Wistar rats were exposed to 5 binges of ethanol (3 g/kg/day); after 24 h of last binge supplementation, for three consecutive days AEGl (100 mg/kg/day) or distilled water were given to animals	Improve brain health	[85]
**Mulberry fruit and ginger**	Combined extract (PMG)	A 16 week feeding study of high-carbohydrate high-fat (Wistar rats). PMG was orally administered 21 days before and 21 days after the occlusion of the right middle cerebral artery to MetS rats at doses of 50, 100, and 200 mg·kg^−1^ BW	Improvement in oxidative stress and brain health	[86]
**Lemon seeds**	Flavonoids	Different flavonoid concentrations (150 μg/mL 100 μg/mL and 50 μg/mL) were used to treat HEK 293T cells	Improvement in oxidative damage	[87]
**Tea**	Gallocatechin gallate	Cholesterol micellar solubility of was determined (Using catechins 1 and 2 mM), Lymphatic Recovery of ^14^C-Cholesterol in Rats Cannulated in the Thoracic Duct performed (using 100 mg and 120 mg in 3 mL catechins emulsion)	Reduction of cholesterol	[88]
**Grape**	Polyphenols	Double-blind, crossover study, 30 days consumption of freeze-dried grape polyphenol powder or a placebo	Reduction of cardiovascular disease	[89]

## Data Availability

Not applicable.

## References

[1] World Health Organization (2019). *Healthy Diet* (No. WHO-EM/NUT/282/E). World Health Organization. Regional Office for the Eastern Mediterranean. https://apps.who.int/iris/bitstream/handle/10665/325828/EMROPUB_2019_en_23536.pdf.

[2] Gürbüz N., Uluişik S., Frary A., Frary A., Doğanlar S. (2018). Health benefits and bioactive compounds of eggplant. Food Chem..

[3] Liu R.H. (2013). Dietary Bioactive Compounds and Their Health Implications. J. Food Sci..

[4] Acosta-Estrada B.A., Gutiérrez-Uribe J.A., Serna-Saldívar S.O. (2014). Bound phenolics in foods, a review. Food Chem..

[5] Šaponjac V.T., Čanadanović-Brunet J., Ćetković G., Djilas S. (2016). Detection of Bioactive Compounds in Plants and Food Products. Emerging and Traditional Technologies for Safe, Healthy and Quality Food.

[6] Prakash B., Kujur A., Singh P.P., Kumar A., Yadav A. (2017). Plants-derived bioactive compounds as functional food ingredients and food preservative. J. Nutr. Food Sci..

[7] Plazas M., López-Gresa M.P., Vilanova S., Torres C., Hurtado M., Gramazio P., Andújar I., Herráiz F.J., Bellés J.M., Prohens J. (2013). Diversity and Relationships in Key Traits for Functional and Apparent Quality in a Collection of Eggplant: Fruit Phenolics Content, Antioxidant Activity, Polyphenol Oxidase Activity, and Browning. J. Agric. Food Chem..

[8] Singh J.P., Kaur A., Shevkani K., Singh N. (2015). Influence of jambolan (Syzygium cumini) and xanthan gum incorporation on the physicochemical, antioxidant and sensory properties of gluten-free eggless rice muffins. Int. J. Food Sci. Technol..

[9] Li Y., Zhang J.-J., Xu D.-P., Zhou T., Zhou Y., Li S., Li H.-B. (2016). Bioactivities and Health Benefits of Wild Fruits. Int. J. Mol. Sci..

[10] Zhang J.-J., Li Y., Zhou T., Xu D.-P., Zhang P., Li S., Li H.-B. (2016). Bioactivities and Health Benefits of Mushrooms Mainly from China. Molecules.

[11] Xu D.-P., Li Y., Meng X., Zhou T., Zhou Y., Zheng J., Zhang J.-J., Li H.-B. (2017). Natural Antioxidants in Foods and Medicinal Plants: Extraction, Assessment and Resources. Int. J. Mol. Sci..

[12] Miguel M., Alonso M.J., Salaices M., Aleixandre A., López-Fandiño R. (2007). Antihypertensive, ACE-inhibitory and vasodilator properties of an egg white hydrolysate: Effect of a simulated intestinal digestion. Food Chem..

[13] Chakrabarti S., Guha S., Majumder K. (2018). Food-Derived Bioactive Peptides in Human Health: Challenges and Opportunities. Nutrient.

[14] Hartmann R., Meisel H. (2007). Food-derived peptides with biological activity: From research to food applications. Curr. Opin. Biotechnol..

[15] Aluko R.E. (2008). Antihypertensive properties of plant-derived inhibitors of angiotensin I-converting enzyme activity: A review. Recent Prog. Med. Plants..

[16] Harnedy P.A., FitzGerald R.J. (2012). Bioactive peptides from marine processing waste and shellfish: A review. J. Funct. Foods.

[17] Cavazos A., De Mejia E.G. (2013). Identification of Bioactive Peptides from Cereal Storage Proteins and Their Potential Role in Prevention of Chronic Diseases. Compr. Rev. Food Sci. Food Saf..

[18] Ahmed F., Fanning K., Netzel M., Turner W., Li Y., Schenk P.M. (2014). Profiling of carotenoids and antioxidant capacity of microalgae from subtropical coastal and brackish waters. Food Chem..

[19] Benke K.K., Benke K.E. (2014). Experimental Design Issue for Assessment of Carotenoids Lutein and Zeaxanthin in Age-Related Eye Disease Study 2 Formulation for Age-Related Macular Degeneration. JAMA Ophthalmol..

[20] Weikel K.A., Garber C., Baburins A., Taylor A. (2014). Nutritional modulation of cataract. Nutr. Rev..

[21] Di Pietro N., Di Tomo P., Pandolfi A. (2016). Carotenoids in cardiovascular disease prevention. JSM Atheroscler..

[22] Cooperstone J., Schwartz S. (2016). Recent Insights into Health Benefits of Carotenoids. Handbook on Natural Pigments in Food and Beverages.

[23] Manach C., Scalbert A., Morand C., Rémésy C., Jiménez L. (2004). Polyphenols: Food sources and bioavailability. Am. J. Clin. Nutr..

[24] Casati L., Pagani F., Braga P.C., Scalzo R.L., Sibilia V. (2016). Nasunin, a new player in the field of osteoblast protection against oxidative stress. J. Funct. Foods.

[25] Khoo H.E., Azlan A., Tang S.T., Lim S.M. (2017). Anthocyanidins and anthocyanins: Colored pigments as food, pharmaceutical ingredients, and the potential health benefits. Food Nutr. Res..

[26] Bennett R.N., Shiga T.M., Hassimotto N.M.A., Rosa E.A.S., Lajolo F.M., Cordenunsi B.R. (2010). Phenolics and Antioxidant Properties of Fruit Pulp and Cell Wall Fractions of Postharvest Banana (Musa acuminata Juss.) Cultivars. J. Agric. Food Chem..

[27] Singh B., Singh J.P., Kaur A., Singh N. (2016). Bioactive compounds in banana and their associated health benefits—A review. Food Chem..

[28] Gropper S.S., Smith J.L. (2012). Advanced Nutrition and Human Metabolism Cengage Learning. https://www.cengage.com/c/advanced-nutrition-and-human-metabolism-6e-gropper/9781133104056PF/.

[29] Kim M.J., Moon Y., Tou J.C., Mou B., Waterland N.L. (2016). Nutritional value, bioactive compounds and health benefits of lettuce (*Lactuca sativa* L.). J. Food Compos. Anal..

[30] Erlund I. (2004). Review of the flavonoids quercetin, hesperetin, and naringenin. Dietary sources, bioactivities, bioavailability, and epidemiology. Nutr. Res..

[31] Liggins J., Bluck L.J.C., Runswick S., Atkinson C., Coward W.A., Bingham S.A. (2000). Daidzein and genistein contents of vegetables. Br. J. Nutr..

[32] D’Andrea G. (2015). Quercetin: A flavonol with multifaceted therapeutic applications?. Fitoterapia.

[33] Ghofrani S., Joghataei M.-T., Mohseni S., Baluchnejadmojarad T., Bagheri M., Khamse S., Roghani M. (2015). Naringenin improves learning and memory in an Alzheimer’s disease rat model: Insights into the underlying mechanisms. Eur. J. Pharmacol..

[34] Pereira-Caro G., Gaillet S., Ordóñez J.L., Mena P., Bresciani L., Bindon K.A., Del Rio D., Rouanet J.-M., Moreno-Rojas J.M., Crozier A. (2020). Bioavailability of red wine and grape seed proanthocyanidins in rats. Food Funct..

[35] Moreno-Ortega A., Ordóñez J.L., Moreno-Rojas R., Moreno-Rojas J.M., Pereira-Caro G. (2021). Changes in the Organosulfur and Polyphenol Compound Profiles of Black and Fresh Onion during Simulated Gastrointestinal Digestion. Foods.

[36] Moreno-Ortega A., Pereira-Caro G., Ordóñez J.L., Muñoz-Redondo J.M., Moreno-Rojas R., Pérez-Aparicio J., Moreno-Rojas J.M. (2020). Changes in the antioxidant activity and metabolite profile of three onion varieties during the elaboration of ‘black onion’. Food Chem..

[37] Pereira-Caro G., Clifford M.N., Polyviou T., Ludwig I.A., Alfheeaid H., Moreno-Rojas J.M., Garcia A.L., Malkova D., Crozier A. (2020). Plasma pharmacokinetics of (poly)phenol metabolites and catabolites after ingestion of orange juice by endurance trained men. Free. Radic. Biol. Med..

[38] Ordóñez-Díaz J.L., Hervalejo A., Pereira-Caro G., Muñoz-Redondo J.M., Romero-Rodríguez E., Arenas-Arenas F.J., Moreno-Rojas J.M. (2020). Effect of Rootstock and Harvesting Period on the Bioactive Compounds and Antioxidant Activity of Two Orange Cultivars (‘Salustiana’ and ‘Sanguinelli’) Widely Used in Juice Industry. Processes.

[39] Moreno-Ortega A., Pereira-Caro G., Ordóñez J.L., Moreno-Rojas R., Ortíz-Somovilla V., Moreno-Rojas J.M. (2020). Bioaccessibility of Bioactive Compounds of ‘Fresh Garlic’ and ‘Black Garlic’ through In Vitro Gastrointestinal Digestion. Foods.

[40] Quintero-Flórez A., Pereira-Caro G., Sánchez-Quezada C., Moreno-Rojas J.M., Gaforio J.J., Jimenez A., Beltrán G. (2018). Effect of olive cultivar on bioaccessibility and antioxidant activity of phenolic fraction of virgin olive oil. Eur. J. Nutr..

[41] Majid A., Priyadarshini C.G.P. (2020). Millet derived bioactive peptides: A review on their functional properties and health benefits. Crit. Rev. Food Sci. Nutr..

[42] Dias-Martins A.M., Pessanha K.L.F., Pacheco S., Rodrigues J.A.S., Carvalho C.W.P. (2018). Potential use of pearl millet (*Pennisetum glaucum* (L.) R. Br.) in Brazil: Food security, processing, health benefits and nutritional products. Food Res. Int..

[43] Udenigwe C.C., Aluko R.E. (2012). Food Protein-Derived Bioactive Peptides: Production, Processing, and Potential Health Benefits. J. Food Sci..

[44] Moreno-Rojas J.M., Moreno-Ortega A., Ordóñez J.L., Moreno-Rojas R., Pérez-Aparicio J., Pereira-Caro G. (2018). Development and validation of UHPLC-HRMS methodology for the determination of flavonoids, amino acids and organosulfur compounds in black onion, a novel derived product from fresh shallot onions (Allium cepa var. aggregatum). LWT.

[45] World Health Organization Cardiovascular Diseases. https://www.who.int/health-topics/cardiovascular-diseases/#tab=tab_1.

[46] Călinoiu L.F., Vodnar D.C. (2018). Whole Grains and Phenolic Acids: A Review on Bioactivity, Functionality, Health Benefits and Bioavailability. Nutrients.

[47] Sanjukta S., Rai A.K. (2016). Production of bioactive peptides during soybean fermentation and their potential health benefits. Trends Food Sci. Technol..

[48] Sharifi-Rad J., Rodrigues C.F., Sharopov F., Docea A.O., Karaca A.C., Sharifi-Rad M., Karıncaoglu D.K., Gülseren G., Özçelik B., Demircan E. (2020). Diet, Lifestyle and Cardiovascular Diseases: Linking Pathophysiology to Cardioprotective Effects of Natural Bioactive Compounds. Int. J. Environ. Res. Public Health.

[49] Cicero A.F.G., Fogacci F., Colletti A. (2017). Food and plant bioactives for reducing cardiometabolic disease risk: An evidence based approach. Food Funct..

[50] Patten G.S., Abeywardena M.Y., Bennett L.E. (2016). Inhibition of Angiotensin Converting Enzyme, Angiotensin II Receptor Blocking, and Blood Pressure Lowering Bioactivity across Plant Families. Crit. Rev. Food Sci. Nutr..

[51] Varzakas T., Zakynthinos G., Verpoort F. (2016). Plant Food Residues as a Source of Nutraceuticals and Functional Foods. Foods.

[52] Barera A., Buscemi S., Monastero R., Caruso C., Caldarella R., Ciaccio M., Vasto S. (2016). β-glucans: Ex vivo inflammatory and oxidative stress results after pasta intake. Immun. Ageing.

[53] Koyama M., Naramoto K., Nakajima T., Aoyama T., Watanabe M., Nakamura K. (2013). Purification and Identification of Antihypertensive Peptides from Fermented Buckwheat Sprouts. J. Agric. Food Chem..

[54] Campos M.R.S., González F.P., Guerrero L.C., Ancona D.B. (2013). Angiotensin I-Converting Enzyme Inhibitory Peptides of Chia (Salvia hispanica) Produced by Enzymatic Hydrolysis. Int. J. Food Sci..

[55] Toufektsian M.-C., De Lorgeril M., Nagy N., Salen P., Donati M.B., Giordano L., Mock H.-P., Peterek S., Matros A., Petroni K. (2008). Chronic Dietary Intake of Plant-Derived Anthocyanins Protects the Rat Heart against Ischemia-Reperfusion Injury. J. Nutr..

[56] Tejada S., Pinya S., Bibiloni M.D.M., Tur J.A., Pons A., Sureda A. (2017). Cardioprotective Effects of the Polyphenol Hydroxytyrosol from Olive Oil. Curr. Drug Targets.

[57] Afshari F., Seraj H., Hashemi Z.S., Timajchi M., Ensiyeh O., Ladan G., Asadi M., Elyasi Z., GanjiBakhsh M. (2017). The Cytotoxic Effects of Eggplant Peel Extract on Human Gastric Adenocarcinoma Cells and Normal Cells. Mod. Med Lab. J..

[58] Shen K.-H., Hung J.-H., Chang C.-W., Weng Y.-T., Wu M.-J., Chen P.-S. (2017). Solasodine inhibits invasion of human lung cancer cell through downregulation of miR-21 and MMPs expression. Chem. Interact..

[59] Montales M.T.E., Rahal O.M., Kang J., Rogers T.J., Prior R.L., Wu X., Simmen R.C. (2012). Repression of mammosphere formation of human breast cancer cells by soy isoflavone genistein and blueberry polyphenolic acids suggests diet-mediated targeting of cancer stem-like/progenitor cells. Carcinogenesis.

[60] Zhao D., Yao C., Chen X., Xia H., Zhang L., Liu H., Jiang X., Dai Y., Liu J. (2013). The Fruits of Maclura pomifera Extracts Inhibits Glioma Stem-Like Cell Growth and Invasion. Neurochem. Res..

[61] Mak K.-K., Wu A.T.H., Lee W.-H., Chang T.-C., Chiou J.-F., Wang L.-S., Wu C.-H., Huang C.-Y.F., Shieh Y.-S., Chao T.-Y. (2013). Pterostilbene, a bioactive component of blueberries, suppresses the generation of breast cancer stem cells within tumor microenvironment and metastasis via modulating NF-κB/microRNA 448 circuit. Mol. Nutr. Food Res..

[62] Leung E.H.W., Ng T.B. (2007). A relatively stable antifungal peptide from buckwheat seeds with antiproliferative activity toward cancer cells. J. Pept. Sci..

[63] Vilcacundo R., Miralles B., Carrillo W., Hernández-Ledesma B. (2018). In vitro chemopreventive properties of peptides released from quinoa (Chenopodium quinoa Willd.) protein under simulated gastrointestinal digestion. Food Res. Int..

[64] Faria A., Pestana D., Teixeira D., De Freitas V., Mateus N., Calhau C. (2010). Blueberry anthocyanins and pyruvic acid adducts: Anticancer properties in breast cancer cell lines. Phytother. Res..

[65] Lee S.-H., Lee J., Herald T., Cox S., Noronha L., Perumal R., Lee H.-S., Smolensky D. (2020). Anticancer Activity of a Novel High Phenolic Sorghum Bran in Human Colon Cancer Cells. Oxidative Med. Cell. Longev..

[66] Cheng D.M., Pogrebnyak N., Kuhn P., Poulev A., Waterman C., Rojas-Silva P., Johnson W.D., Raskin I. (2014). Polyphenol-rich Rutgers Scarlet Lettuce improves glucose metabolism and liver lipid accumulation in diet-induced obese C57BL/6 mice. Nutrients.

[67] Vilcacundo R., Martínez-Villaluenga C., Hernández-Ledesma B. (2017). Release of dipeptidyl peptidase IV, α-amylase and α-glucosidase inhibitory peptides from quinoa (Chenopodium quinoa Willd.) during in vitro simulated gastrointestinal digestion. J. Funct. Foods.

[68] Yang H.J., Kim H.J., Kim M.J., Kang S., Kim D.S., Daily J.W., Jeong D.Y., Kwon D.Y., Park S. (2012). Standardized chungkookjang, short-term fermented soybeans with Bacillus lichemiformis, improves glucose homeostasis as much as traditionally made chungkookjang in diabetic rats. J. Clin. Biochem. Nutr..

[69] Li D., Zhang Y., Liu Y., Sun R., Xia M. (2015). Purified Anthocyanin Supplementation Reduces Dyslipidemia, Enhances Antioxidant Capacity, and Prevents Insulin Resistance in Diabetic Patients. J. Nutr..

[70] Zhang H., Wang J., Liu Y., Sun B. (2015). Peptides Derived from Oats Improve Insulin Sensitivity and Lower Blood Glucose in Streptozotocin-Induced Diabetic Mice. J. Biomed. Sci..

[71] Wang J., Du K., Fang L., Liu C., Min W., Liu J. (2018). Evaluation of the antidiabetic activity of hydrolyzed peptides derived from Juglans mandshurica Maxim. fruits in insulin-resistant HepG2 cells and type 2 diabetic mice. J. Food Biochem..

[72] Barik S.K., Russell W.R., Moar K.M., Cruickshank M., Scobbie L., Duncan G., Hoggard N. (2020). The anthocyanins in black currants regulate postprandial hyperglycaemia primarily by inhibiting α-glucosidase while other phenolics modulate salivary α-amylase, glucose uptake and sugar transporters. J. Nutr. Biochem..

[73] Zhang X., Zhu X., Sun Y., Hu B., Sun Y., Jabbar S., Zeng X. (2013). Fermentation in vitro of EGCG, GCG and EGCG3”Me isolated from Oolong tea by human intestinal microbiota. Food Res. Int..

[74] Chacar S., Itani T., Hajal J., Saliba Y., Louka N., Faivre J.-F., Maroun R., Fares N. (2018). The Impact of Long-Term Intake of Phenolic Compounds-Rich Grape Pomace on Rat Gut Microbiota. J. Food Sci..

[75] Han M., Song P., Huang C., Rezaei A., Farrar S., Brown M.A., Ma X. (2016). Dietary grape seed proanthocyanidins (GSPs) improve weaned intestinal microbiota and mucosal barrier using a piglet model. Oncotarget.

[76] Anhê F.F., Roy D., Pilon G., Dudonné S., Matamoros S., Varin T.V., Garofalo C., Moine Q., Desjardins Y., Levy E. (2015). A polyphenol-rich cranberry extract protects from diet-induced obesity, insulin resistance and intestinal inflammation in association with increased Akkermansia spp. population in the gut microbiota of mice. Gut.

[77] Nagata R., Echizen M., Yamaguchi Y., Han K.-H., Shimada K., Ohba K., Kitano-Okada T., Nagura T., Uchino H., Fukushima M. (2018). Effect of a combination of inulin and polyphenol-containing adzuki bean extract on intestinal fermentation in vitro and in vivo. Biosci. Biotechnol. Biochem..

[78] Kim J., Choi J.H., Ko G., Jo H., Oh T., Ahn B., Unno T. (2020). Anti-Inflammatory Properties and Gut Microbiota Modulation of *Porphyra tenera* Extracts in Dextran Sodium Sulfate-Induced Colitis in Mice. Antioxidants.

[79] Basuny A.M., Arafat S.M., El-Marzooq M.A. (2012). Antioxidant and antihyperlipidemic activities of anthocyanins from egg-plant peels. J. Pharma Res. Rev..

[80] Vijayakumar S., Presannakumar G., Vijayalakshmi N.R. (2009). Investigations on the Effect of Flavonoids from Banana, *Musa Paradisiaca* L. on Lipid Metabolism in Rats. J. Diet. Suppl..

[81] Halaby M.S., Metwally E.M., Omar A.A. (2013). Effect of Moringa oleifera on serum lipids and kidney function of hyperlipidemic rats. J. Appl. Sci. Res..

[82] Neyrinck A.M., Van Hée V.F., Bindels L.B., De Backer F., Cani P., Delzenne N.M. (2013). Polyphenol-rich extract of pomegranate peel alleviates tissue inflammation and hypercholesterolaemia in high-fat diet-induced obese mice: Potential implication of the gut microbiota. Br. J. Nutr..

[83] Li N., Sun Y.-R., He L.-B., Huang L., Li T.-T., Wang T.-Y., Tang L. (2020). Amelioration by Idesia polycarpa Maxim. var. vestita Diels. of Oleic Acid-Induced Nonalcoholic Fatty Liver in HepG2 Cells through Antioxidant and Modulation of Lipid Metabolism. Oxidative Med. Cell. Longev..

[84] Giacoppo S., Rajan T.S., De Nicola G.R., Iori R., Rollin P., Bramanti P., Mazzon E. (2017). The Isothiocyanate Isolated from Moringa oleifera Shows Potent Anti-Inflammatory Activity in the Treatment of Murine Subacute Parkinson’s Disease. Rejuvenation Res..

[85] Nascimento C.P., Luz D.A., da Silva C.C.S., Malcher C.M.R., Fernandes L.M.P., Santa H.S.D., Gomes A.R.Q., Monteiro M.C., Ribeiro C.H.M.A., Fontes-Júnior E.A. (2020). Ganoderma lucidum Ameliorates Neurobehavioral Changes and Oxidative Stress Induced by Ethanol Binge Drinking. Oxidative Med. Cell. Longev..

[86] Wattanathorn J., Palachai N., Thukham-Mee W., Muchimapura S. (2020). Memory-Enhancing Effect of a Phytosome Containing the Combined Extract of Mulberry Fruit and Ginger in an Animal Model of Ischemic Stroke with Metabolic Syndrome. Oxidative Med. Cell. Longev..

[87] Yang D., Jiang Y., Wang Y., Lei Q., Zhao X., Yi R., Zhang X. (2020). Improvement of Flavonoids in Lemon Seeds on Oxidative Damage of Human Embryonic Kidney 293T Cells Induced by H_2_O_2_. Oxidative Med. Cell. Longev..

[88] Ikeda I., Kobayashi M., Hamada T., Tsuda K., Goto H., Imaizumi K., Nozawa A., Sugimoto A., Kakuda T. (2003). Heat-Epimerized Tea Catechins Rich in Gallocatechin Gallate and Catechin Gallate are More Effective to Inhibit Cholesterol Absorption than Tea Catechins Rich in Epigallocatechin Gallate and Epicatechin Gallate. J. Agric. Food Chem..

[89] Barona J., Aristizabal J.C., Blesso C.N., Volek J.S., Fernandez M.L. (2012). Grape Polyphenols Reduce Blood Pressure and Increase Flow-Mediated Vasodilation in Men with Metabolic Syndrome. J. Nutr..

[90] Pistollato F., Giampieri F., Battino M. (2015). The use of plant-derived bioactive compounds to target cancer stem cells and modulate tumor microenvironment. Food Chem. Toxicol..

[91] Li Y., Wicha M.S., Schwartz S.J., Sun D. (2011). Implications of cancer stem cell theory for cancer chemoprevention by natural dietary compounds. J. Nutr. Biochem..

[92] Tajik N., Tajik M., Mack I., Enck P. (2017). The potential effects of chlorogenic acid, the main phenolic components in coffee, on health: A comprehensive review of the literature. Eur. J. Nutr..

[93] Berghe W.V. (2012). Epigenetic impact of dietary polyphenols in cancer chemoprevention: Lifelong remodeling of our epigenomes. Pharmacol. Res..

[94] Prasad S., Phromnoi K., Yadav V.R., Chaturvedi M.M., Aggarwal B.B. (2010). Targeting Inflammatory Pathways by Flavonoids for Prevention and Treatment of Cancer. Planta Med..

[95] Salminen A., Lehtonen M., Suuronen T., Kaarniranta K., Huuskonen J. (2008). Terpenoids: Natural inhibitors of NF-κB signaling with anti-inflammatory and anticancer potential. Cell. Mol. Life Sci..

[96] Dobani S., Latimer C., McDougall G.J., Allwood J.W., Pereira-Caro G., Moreno-Rojas J.M., Ternan N.G., Pourshahidi L.K., Lawther R., Tuohy K.M. (2021). Ex vivo fecal fermentation of human ileal fluid collected after raspberry consumption modifies (poly)phenolics and modulates genoprotective effects in colonic epithelial cells. Redox Biol..

[97] Henidi H.A., Al-Abbasi F.A., El-Moselhy M.A., El-Bassossy H.M., Al-Abd A.M. (2020). Despite Blocking Doxorubicin-Induced Vascular Damage, Quercetin Ameliorates Its Anti breast Cancer Activity. Oxidative Med. Cell. Longev..

[98] Patil S.P., Goswami A., Kalia K., Kate A.S. (2020). Plant-Derived Bioactive Peptides: A Treatment to Cure Diabetes. Int. J. Pept. Res. Ther..

[99] Gong X., Ji M., Xu J., Zhang C., Li M. (2020). Hypoglycemic effects of bioactive ingredients from medicine food homology and medicinal health food species used in China. Crit. Rev. Food Sci. Nutr..

[100] Yang L., Shu L., Yao D.D., Jia X.B., Yu S.M. (2014). Study on the glucose-lowering effect of puerarin in STZ-induced diabetic mice. Chin. J. Hosp. Pharm..

[101] Wan M.L.Y., Ling K.H., El-Nezami H., Wang M.F. (2019). Influence of functional food components on gut health. Crit. Rev. Food Sci. Nutr..

[102] Bischoff S.C., Barbara G., Buurman W., Ockhuizen T., Schulzke J.-D., Serino M., Tilg H., Watson A., Wells J.M. (2014). Intestinal permeability—A new target for disease prevention and therapy. BMC Gastroenterol..

[103] Hidalgo-Liberona N., González-Domínguez R., Vegas E., Riso P., Del Bo’ C., Bernardi S., Peron G., Guglielmetti S., Gargari G., Kroon P.A. (2020). Increased Intestinal Permeability in Older Subjects Impacts the Beneficial Effects of Dietary Polyphenols by Modulating Their Bioavailability. J. Agric. Food Chem..

[104] Loo Y.T., Howell K., Chan M., Zhang P., Ng K. (2020). Modulation of the human gut microbiota by phenolics and phenolic fiber-rich foods. Compr. Rev. Food Sci. Food Saf..

[105] Dey P. (2019). Gut microbiota in phytopharmacology: A comprehensive overview of concepts, reciprocal interactions, biotransformations and mode of actions. Pharmacol. Res..

[106] Gong L., Cao W., Chi H., Wang J., Zhang H., Liu J., Sun B. (2018). Whole cereal grains and potential health effects: Involvement of the gut microbiota. Food Res. Int..

[107] Siasos G., Tousoulis D., Tsigkou V., Kokkou E., Oikonomou E., Vavuranakis M., Basdra E., Papavassiliou A., Stefanadis C. (2013). Flavonoids in Atherosclerosis: An Overview of Their Mechanisms of Action. Curr. Med. Chem..

[108] Vergara-Jimenez M., AlMatrafi M.M., Fernandez M.L. (2017). Bioactive Components in Moringa Oleifera Leaves Protect against Chronic Disease. Antioxidants.

[109] Akram N.A., Shafiq F., Ashraf M. (2018). Peanut (*Arachis hypogaea* L.): A Prospective Legume Crop to Offer Multiple Health Benefits under Changing Climate. Compr. Rev. Food Sci. Food Saf..

[110] Marangoni F., Poli A. (2010). Phytosterols and cardiovascular health. Pharmacol. Res..

[111] Galvez A.F.U.S. (2013). Products and Methods Using Soy Peptides to Lower Total and LDL Cholesterol Levels.

[112] Xu J., Ma C., Han L., Gao H., Zhou Q., Yang M., Chen C., Deng Q., Huang Q., Huang F. (2014). Optimized rapeseed oils rich in endogenous micronutrients ameliorate risk factors of atherosclerosis in high fat diet fed rats. Lipids Health Dis..

[113] Welcome M.O. (2020). Neuroinflammation in CNS diseases: Molecular mechanisms and the therapeutic potential of plant derived bioactive molecules. PharmaNutrition.

[114] Piemontese L. (2017). Plant Food Supplements with Antioxidant Properties for the Treatment of Chronic and Neurodegenerative Diseases: Benefits or Risks?. J. Diet. Suppl..

[115] Lu M., Tan L., Zhou X.-G., Yang Z.-L., Zhu Q., Chen J.-N., Luo H.-R., Wu G.-S. (2020). Secoisolariciresinol Diglucoside Delays the Progression of Aging-Related Diseases and Extends the Lifespan of Caenorhabditis elegans via DAF-16 and HSF-1. Oxidative Med. Cell. Longev..

[116] Libro R., Giacoppo S., Rajan T.S., Bramanti P., Mazzon E. (2016). Natural Phytochemicals in the Treatment and Prevention of Dementia: An Overview. Molecules.

[117] Regitz C., Fitzenberger E., Mahn F.L., Dußling L.M., Wenzel U. (2016). Resveratrol reduces amyloid-beta (Aβ1–42)-induced paralysis through targeting proteostasis in an Alzheimer model of Caenorhabditis elegans. Eur. J. Nutr..

[118] Vijayakumar S., Presannakumar G., Vijayalakshmi N. (2008). Antioxidant activity of banana flavonoids. Fitoterapia.

[119] Pereira-Caro G., Polyviou T., Ludwig I.A., Nastase A.-M., Moreno-Rojas J.M., Garcia A.L., Malkova D., Crozier A. (2017). Bioavailability of orange juice (poly)phenols: The impact of short-term cessation of training by male endurance athletes. Am. J. Clin. Nutr..

[120] Quesada-Gómez J.M., Santiago-Mora R., Durán-Prado M., Dorado G., Pereira-Caro G., Moreno-Rojas J.M., Casado-Díaz A. (2017). β-Cryptoxanthin Inhibits Angiogenesis in Human Umbilical Vein Endothelial Cells Through Retinoic Acid Receptor. Mol. Nutr. Food Res..

[121] Sagar N.A., Pareek S., Sharma S., Yahia E.M., Lobo M.G. (2018). Fruit and Vegetable Waste: Bioactive Compounds, Their Extraction, and Possible Utilization. Compr. Rev. Food Sci. Food Saf..

[122] Gülçin I. (2012). Antioxidant activity of food constituents: An overview. Arch. Toxicol..

[123] Leuci R., Brunetti L., Poliseno V., Laghezza A., Loiodice F., Tortorella P., Piemontese L. (2021). Natural Compounds for the Pre-vention and Treatment of Cardiovascular and Neurodegenerative Diseases. Foods.

[124] Dehelean C.A., Marcovici I., Soica C., Mioc M., Coricovac D., Iurciuc S., Cretu O.M., Pinzaru I. (2021). Plant-Derived Anti-cancer Compounds as New Perspectives in Drug Discovery and Alternative Therapy. Molecules.

[125] Noce A., Di Lauro M., Di Daniele F., Zaitseva A.P., Marrone G., Borboni P., Di Daniele N. (2021). Natural Bioactive Compounds Useful in Clinical Management of Metabolic Syndrome. Nutrients.

